# Ventricular global function index is associated with clinical outcomes in pediatric pulmonary hypertension

**DOI:** 10.1186/s12968-023-00947-8

**Published:** 2023-07-03

**Authors:** Hieu T. Ta, Paul J. Critser, Michal Schäfer, Nicholas J. Ollberding, Michael D. Taylor, Michael V. Di Maria, Russel Hirsch, D. Dunbar Ivy, Benjamin S. Frank

**Affiliations:** 1 Cincinnati Children’s Hospital Medical Center, Cincinnati, OH USA; 2 Department of Pediatrics, College of Medicine, University of Cincinnati, Cincinnati, OH USA; 3 Department of Pediatrics Section of Cardiology, University of Colorado, Aurora, CO USA; 4 Division of Biostatistics and Epidemiology, Cincinnati Children’s Hospital Medical Center, Cincinnati, OH USA

**Keywords:** Pediatric pulmonary hypertension, Global function index, Cardiovascular magnetic resonance

## Abstract

**Background:**

Multiple right ventricular (RV) metrics have prognostic value in pulmonary hypertension (PH). A cardiac magnetic resonance imaging (CMR) derived global ventricular function index (GFI) provided improved prediction of composite adverse outcome (CAO) in adults with atherosclerosis. GFI has not yet been explored in a PH population. We explored the feasibility of GFI as a predictor of CAO in a pediatric PH population.

**Methods:**

Two center retrospective chart review identified pediatric PH patients undergoing CMR from Jan 2005–June 2021. GFI, defined as the ratio of the stroke volume to the sum of mean ventricular cavity and myocardial volume, was calculated for each patient. CAO was defined as death, lung transplant, Potts shunt, or parenteral prostacyclin initiation after CMR. Cox proportional hazards regression was used to estimate associations and assess model performance between CMR parameters and CAO.

**Results:**

The cohort comprised 89 patients (54% female, 84% World Health Organization (WHO) Group 1; 70% WHO-FC ≤ 2; and 27% on parenteral prostacyclin). Median age at CMR was 12 years (IQR 8.1–17). Twenty-one (24%) patients experienced CAO during median follow up of 1.5 years. CAO cohort had higher indexed RV volumes (end systolic—145 vs 99 mL/m^2^, p = 0.003; end diastolic—89 vs 46 mL/m^2^, p = 0.004) and mass (37 vs 24 gm/m^2^, p = 0.003), but lower ejection fraction (EF) (42 vs 51%, p < 0.001) and GFI (40 vs 52%, p < 0.001). Higher indexed RV volumes (hazard ratios [HR] 1.01, CI 1.01–1.02), lower RV EF (HR 1.09, CI 1.05–1.12) and lower RV GFI (HR 1.09, CI 1.05–1.11) were associated with increased risk of CAO. In survival analysis, patients with RV GFI < 43% demonstrated decreased event-free survival and increased hazard of CAO compared to those with RV GFI ≥ 43%. In multivariable models, inclusion of GFI provided improved prediction of CAO compared to models incorporating ventricular volumes, mass or EF.

**Conclusions:**

RV GFI was associated with CAO in this cohort, and inclusion in multivariable models had increased predictive value compared to RVEF. GFI uses readily available CMR data without additional post-processing and may provide additional prognostic value in pediatric PH patients beyond traditional CMR markers.

**Supplementary Information:**

The online version contains supplementary material available at 10.1186/s12968-023-00947-8.

## Main text

### Background

Pediatric pulmonary hypertension (PH) is a progressive condition that can be associated with a variety of cardiac, pulmonary, and systemic diseases, that despite advances in treatment causes significant morbidity and mortality [1, 2]. There is ongoing research into various imaging biomarkers that can be predictive of adverse outcomes in the PH population. Of particular interest has been the assessment of right ventricular (RV) health whether by echocardiography or cardiac MRI (CMR) and its association with outcomes. Diminished function as assessed by decreased stroke volume [3], ejection fraction (EF) [4, 5], or fractional area change [6] has been associated with worse clinical outcomes. RV dilation has also been shown to be a predictor of mortality or treatment failure [3]. Furthermore, RV hypertrophy, which can either be adaptive or mal-adaptive, has also been associated with outcomes [3, 7]. Acknowledging that EF, as an assessment of function, has to be interpreted in the context of preload and afterload [8], a CMR-derived ventricular global function index (GFI) has been proposed as a marker of ventricular health, integrating structural, mechanical, and preload indices [9]. In a cohort of adult patients with atherosclerosis, GFI provided improved prediction of composite adverse outcomes (CAO) compared with EF [9, 10]. In patients with congenital heart disease, GFI has been associated with exercise capacity in repaired tetralogy of Fallot [11] and Fontan patients [12]. In the Fontan cohort, GFI showed an association with Fontan failure while EF did not [12]. However, no studies have assessed the relationship between GFI and CAO in the pediatric PH population.

We sought to evaluate GFI as a predictor of clinical outcomes in the pediatric PH population. Our primary aim was to demonstrate the relationship between RV GFI and incidence of CAO defined as death, lung transplant, Potts shunt, or parenteral prostacyclin initiation (PCA). Our secondary aim was to explore associations between GFI with the following outcomes—six minute walk test (6MWT) distance and natriuretic peptide levels (NT-proBNP). We hypothesized that patients with lower GFI would have increased risk of CAO, lower 6MWT distance, and higher NT-proBNP levels.

### Methods

#### Study design

Data for this study were obtained from a retrospective review of patient medical records from two centers (Children’s Hospital Colorado [CHCO] and Cincinnati Children’s Hospital Medical Center [CCHMC]) and was approved by the institutional review boards of each institution.

#### Patient population

The study cohort consisted of pediatric PH patients who underwent CMR from Jan 2005–June 2021. PH was defined as mean pulmonary artery pressure ≥ 20 mmHg, and pulmonary vascular resistance ≥ 3 iWU. All subjects were followed by PH experts at each institution. PH patients in WHO Group 1, 3, and 5 were included. PH patients with complex congenital heart disease, including single ventricle physiology, (WHO Group 5.4) were excluded. Patients with WHO Group 1 and additional group diagnoses were categorized solely into the WHO Group 1 cohort for analysis. WHO functional class (WHO-FC) was recorded from chart review. 6MWT and NT-proBNP levels were recorded if tests were performed within 6 months of CMR. If obtained, patients with a 6MWT were dichotomized to those with distance < 352 m; distance chosen a priori as a marker of high risk [13–15].

#### CMR acquisition and analysis

CMR studies were performed on either 1.5 Tesla Philips Ingenia scanners (CCHMC/CHCO) or 3 Tesla Philips Ingenia scanners (CHCO). Cardiac functional imaging was performed using a standard retrospective ECG-gated, segmented steady state free precession technique and included a short axis stack of cine steady state free precession images from the cardiac base to apex as previously described [16]. Scan parameters included 6 mm slice thickness with no gap; 1.5 mm^2^ acquired in-plane resolution; field of view manipulated to maintain constant resolution for body size; 30 phases/RR interval; minimum TE; TR ≈2.8 ms [17]. Post processing was performed by manually drawing contours at the endocardial and epicardial borders from short axis cine stack images spanning the ventricular base to apex using CVI42 (Circle cardiovascular Imaging Inc., Calgary, Canada).

If multiple CMRs were performed during the study period, the most recent CMR prior to last follow up or first CAO was used for data collection and statistical analysis. CMR indices, such as ventricular volumes, stroke volume, ejection fraction, and RV mass, were collected via chart review.

Biventricular GFI was calculated for each patient using the following formula as described previously [9]:

$$GFI= \left[\frac{End \, diastolic \,volume \left(EDV\right)-End \, systolic \, volume \, (ESV)}{\frac{EDV+ESV}{2}+ \frac{Ventricular \, mass}{1.05}}\right] x 100$$

If pulmonary regurgitation was more than mild (regurgitant fraction greater than 10%), a modified GFI (eGFI) was calculated in the following way as previously described [11]:

$$RV eGFI=\left[\frac{\left(EDV-ESV\right)x (1-pulmonary \, regurgitant \, fraction)}{\frac{EDV+ESV}{2}+ \frac{Ventricular \, mass}{1.05}}\right]x 100$$

#### Statistical analysis

Patient demographic and clinical characteristics were described using means with standard deviations (or medians and interquartile ranges) and frequencies with percentages. Independent samples t-tests or Fisher’s exact tests were used to test for differences in WHO group, WHO-FC, and PH medications, while Wilcoxon tests were used to test for differences in 6MWT distance, NT-proBNP levels, and CMR indices between those who experienced a CAO and those who did not. Cox proportional hazards regression was used to estimate hazard ratios (HR) and 95% confidence intervals (CI) for the time to CAO. The same statistical analyses were then repeated comparing those who experienced mortality during the study, regardless of if first adverse event, and those who did not. An additional sub-analysis that only included WHO group 1 patients was performed comparing those who experienced CAO versus those who did not. Logistic regression was performed on the entire cohort, and the Youden index was used to determine optimal RV GFI cut off value to predict incidence of CAO. Patients were then dichotomized based on this cut off for survival analysis via a Kaplan–Meier model. Follow-up time accrued from the date of the CMR to the first occurrence of the CAO or date of last follow-up. Patients not experiencing a CAO were censored on the date of last follow-up. Visual inspection of the scaled Schoenfeld residuals plotted against follow time was used to assess the assumption of proportional hazards. Bootstrap resampling (n = 500 resamples) as implemented by the rms package (version 6.2.0) was used to obtain optimism corrected values for the c-statistic. Likelihood ratio tests (LRT) were used to assess improvements in model fit for nested models. Pearson correlations were used to correlate GFI with secondary outcomes. For multivariable modeling, two baseline models were created. The first baseline, “CMR,” model incorporated traditional CMR metrics such as RV volumes and mass. To this CMR baseline model, three derivative models were created by adding either RV CI, RV EF, or RV GFI to assess additive benefits of these markers of RV systolic function. A second baseline, “clinical,” model incorporated a priori clinical factors of WHO-FC, usage of endothelin receptor antagonist (ERA) (which is used as the second agent for patients in our centers), and usage of parenteral PCA. Similarly, three derivative models were created from this clinical model by adding either RV CI, RV EF, or RV GFI. Comparison between models was analyzed by concordance statistic value and LRT. Statistical analysis performed using JMP Pro (Version 16.1, Cary, NC, USA) and the R environment for statistical computing and graphics (version 4.1.1).

### Results

#### Demographics, PH therapy, baseline CMR

The cohort comprised 89 patients (Table 1). Fifty-four percent of the cohort was female. Eighty-four percent were WHO Group 1; 15% were WHO Group 3, and 1% were WHO Group 5. Etiology of PH for WHO Group 3 patients included: bronchopulmonary dysplasia (5); congenital diaphragmatic hernia (4); restrictive lung disease, bronchiectasis (1); interstitial lung disease (1); and congenital cystic adenomatoid malformation (1). The etiology for the patient in WHO Group 5 was acute myeloid leukemia status post bone marrow transplantation. There were no demographic or disease severity differences between etiologic groups. From a functional standpoint, 70% were WHO-FC ≤ 2 (Table 1). Fifty-eight (65%) patients had a 6MWT performed within 6 months of CMR, and median distance was 498 m (interquartile range (IQR): 394–569) (Table 1). A higher percentage of patients with 6MWT performed within 6 months were on ERA therapy (76 vs 45%, p = 0.002) or SQ/IV PCA (34 vs 13%, p = 0.04). Fifty-eight (65%) patients had an NT-proBNP level drawn within 6 months of CMR, and median level was 191 pg/mL (IQR 63–699) (Table 1). A higher percentage of patients with NT-proBNP assessed within 6 months of CMR were on phosphodiesterase-5 inhibitor (PDE-5) therapy (88 vs 71%, P = 0.08) or ERA therapy (74 vs 45%, p = 0.01). The most common PH medication was PDE-5 (73 patients, 82%) followed by ERA (57 patients, 64%), and parenteral PCA (24 patients, 27%). Thirty-four (38%) of patients were on triple therapy; 31 (35%) patients were dual therapy; 24 (27%) were on monotherapy. All patients were on stable therapy prior to CMR. Median age at CMR was 12 years (IQR 8–17) (Table 2). Median RV end-diastolic volume indexed to body surface area (EDVi) was 109 mL/m^2^ (IQR 89–135); median EF was 49% (IQR 43–56); and median RV cardiac index was 4.2 L/min/m^2^ (IQR 3.4–4.9) (Table 2). Two (2%) patients had a pulmonary regurgitant fraction ≥ 10%.

**Table 1:** Demographic data for study cohort and sub-cohorts

	Cohort (N = 89)	CAO (N = 21)	No CAO (N = 68)	p-value
Female n (%)	48 (54)	9 (43)	39 (57)	0.31
*WHO Group*				0.37
1 n (%)	75 (84%)	20 (95)	55 (81)	
3 n (%)	13 (15%)	1 (5)	12 (18)	
5 n (%)	1 (1%)	0 (0)	1 (1)	
*WHO FC*				**0.003**
N (%)	86 (97)			
1 or 2	60 (70)	8 (40)	52 (79)	
3 or 4	26 (30)	12 (60)	14 (21)	
*6MWT (m, N = 58)*				
n (%)	58 (65)	19 (90)	39 (57)	
Meters (IQR)	498 (394–569)	395 (330–530)	535 (430–574)	**0.028**
*NT-proBNP*				
n (%)	58 (65)	16 (76)	42 (62)	
pg/mL (IQR)	191 (63–699)	496 (194–1997)	153 (57–409)	**0.014**
*PH Meds n (%)*				
CCB	9 (10)	1 (5)	8 (12)	0.68
PDE5	73 (82)	19 (90)	54 (79)	0.34
ERA	57 (64)	19 (90)	38 (56)	**0.004**
PO PCA	16 (18)	3 (15)	13 (19)	0.75
SQ/IV PCA	24 (27)	13 (62)	11 (16)	** < 0.001**
Dual therapy	31 (35)	8 (38)	23 (34)	
Triple therapy	34 (38)	13 (62)	21 (24)	**0.019**

Bold indicates p < 0.05

Data are median (Interquartile range). P-values obtained from independent samples t or Fisher’s exact test between CAO and no CAO groups. *CAO* composite adverse outcomes, *CCB* calcium channel blocker, *ERA* endothelin receptor antagonist, *FC* functional class, *IQR* interquartile range, *PCA* prostacyclin, *PDE5* phosphodiesterase-5 inhibitor, *PH* pulmonary hypertension, *PO* oral, *SQ/IV* parenteral

**Table 2:** CMR Data for cohort and sub-cohorts

	Cohort (N = 89)	CAO (N = 21)	No CAO (N = 68)	p-value
Age at CMR (years, IQR)	12.0 (8.0–17.0)	12.0 (10.0–17.0)	12.0 (6.2–16.7)	0.29
*RV*				
RV EDVi (mL/m^2^)	109 (89–135)	145 (119–242)	99 (82–119)	**0.003**
RV ESVi (mL/m^2^)	55 (40–77)	89 (62–195)	46 (37–65)	**0.004**
RV SVi (mL/m^2^)	51 (42–61)	55 (43–63)	51 (42–59)	0.20
RV Mass index (gm/m^2^)	28 (19–37)	37 (31–59)	24 (19–30)	**0.003**
RV EF (%)	49 (43–56)	42 (21–47)	51 (46–56)	** < 0.0001**
RV CI (L/min/m^2^)	4.2 (3.4–4.9)	4.7 (3.6–5.6)	3.9 (3.3–5.6)	0.10
RV GFI (%)	50 (42–57)	40 (18–45)	52 (46–60)	** < 0.001**
*LV*				
LV EDVi, (mL/m^2^)	83 (70–96)	89 (69–117)	81 (70–93)	0.10
LV ESVi (mL/m^2^)	35 (27–44)	35 (28–53)	35 (27–42)	0.10
LV SVi (mL/m^2^)	47 (40–54)	44 (38–56)	47 (40–52)	0.90
LV Mass index (gm/m^2^)	42 36–54)	45 (40–58)	41 (35–49)	0.07
LV EF (%)	56 54–61)	57 (51–62)	56 (54–61)	0.20
LV CI (L/min/m^2^)	3.7 (3.0–4.5)	4.0 (3.1–5.3)	3.6 (3.0–4.3)	0.20
LV GFI (%)	46 (41–51)	43 (37–48)	47 (42–52)	**0.028**

Bold indicates p < 0.05

Data are median (Interquartile range). P values represent Wilcoxon rank-sum test between CAO and no CAO groups. *CAO* composite adverse outcome, *CMR* cardiac magnetic resonance imaging, *CI*  cardiac index, *EDVi*  indexed end diastolic volume, *EF*  ejection fraction, *ESVi*  indexed end systolic volume, *GFI*  global function index, *I*  indexed, *LV*  left ventricle, *RV*  right ventricle, *SVi*  indexed stroke volume

#### CAO

**Fig. 1:**
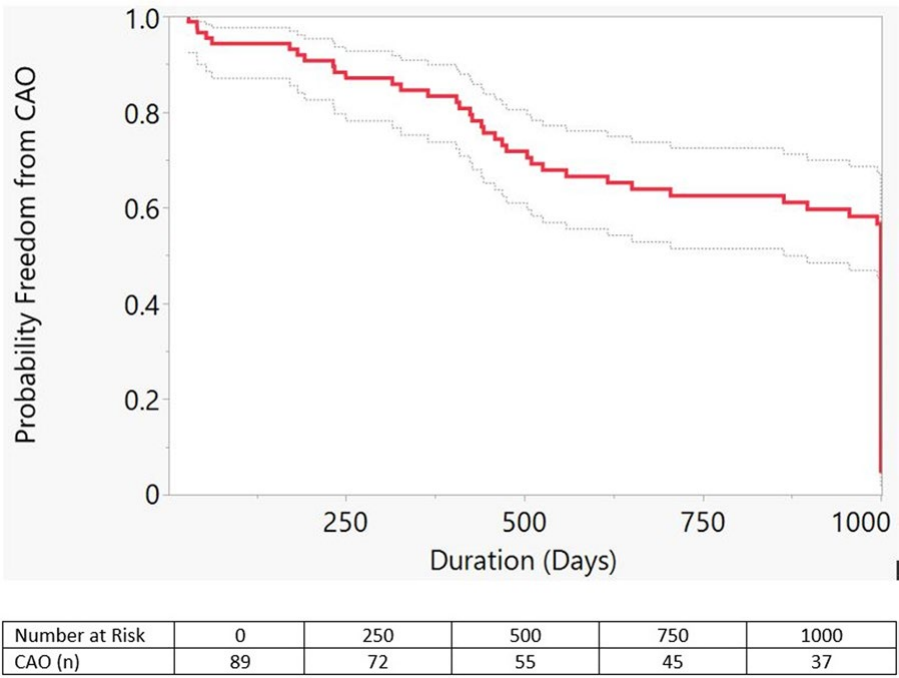
Kaplan–Meier curve demonstrating probability of freedom from composite adverse outcome (CAO: death, lung transplant, Potts shunt, or SQ/IV prostacyclin initiation) for the total population over 1000 days (median follow-up time 710 days). Dashed lines represent 95% confidence intervals (p < 0.0001)

Twenty-one (24%) patients experienced CAO during median follow up of 1.5 years (Fig. 1). Mortality was the second most common outcome (6 patients, 29%) after PCA initiation (8 patients, 38%; Table 3).

**Table 3:** CAO sub-types and time to event

	Total	Death	Lung transplant	Potts shunt	SQ/IV PCA
N (%)	21 (24)	6 (29)	4 (19)	3 (14)	8 (38)
Age (years, IQR)	14.5 (10–19)	18.5 (14.7–22.5)	17 (11–18.8)	12 (9–16)	11.5 (10–17.8)
TTE (years, IQR)	1.5 (0.5–2.5)	1.7 (0.4–3.8)	0.6 (0.3–7.0)	0.7 (0.3–1.8)	1.8 (0.9–2.3)

Data are median (interquartile range). *CAO* composite adverse outcome, *PCA* prostacyclin, *SQ/IV* parenteral, *TTE* time to event

For the CAO sub-cohort, the median age at time of CAO was 14.5 years, and median time to event was 1.5 years after CMR. For those without a CAO, median age at last follow up was 15 years (IQR 11–19), and median follow up time was 1.9 years (IQR 0.9–3.7).

#### CAO vs no CAO

The CAO cohort had higher percentage of patients with WHO-FC ≥ 3 (60% vs 21%, p = 0.003), decreased 6MWT distance (395 vs. 535 m, p = 0.028), and higher NT-proBNP levels (496 vs. 153 pg/mL, p = 0.014) (Table 1). The CAO cohort had a larger percentage of patients on ERA (90 vs 56%, p = 0.004) and/or SQ/IV PCA (62 vs 16%, p < 0.001) (Table 1). The CAO cohort had a larger percentage of patients on triple therapy (62 vs 24%, p = 0.019) (Table 1). From a CMR perspective, the CAO cohort had higher RVEDVi (p = 0.004), RV end systolic volumes indexed to body surface (ESVi) (p = 0.003), and RV mass indexed to body surface area (p = 0.003). The CAO cohort had lower RV EF (p < 0.001), RV GFI (p < 0.001), and LV GFI (p = 0.028) (Table 2). There were no other statistically significant differences at this sample size between the two cohorts for other CMR volumetric and functional analyses.

#### CMR associations with CAO

RV EDVi (HR = 1.01, 95% CI [1.01, 1.02]); ESVi (HR = 1.01, 95% CI [1.01, 1.02]), indexed mass (HR = 1.04, 95% CI [1.03, 1.06)), LV ESVi (HR = 1.03, 95% CI [1.01, 1.05]), and LV indexed mass (HR = 1.03, 1.01, 1.05]) were positively associated with the time to CAO (Table 4). Conversely, RV EF (HR = 0.92, 95% CI [0.89, 0.95]), RV GFI (HR = 0.92, 95% CI [0.90, 0.95]), LV EF (HR = 0.94, 95% CI [0.90, 0.99]), and LV GFI (HR = 0.93, 95% CI [0.88, 0.98]) were inversely associated with the time to CAO. Interestingly, an increase in RV CI was associated with an increased hazard ratio for CAO (HR 1.4, 95% CI [1.0, 1.9]).

**Table 4:** Univariate Cox analysis with CAO

Univariate Cox analysis	Hazard ratio (95% CI)	p value
Gender		0.49
Age at MRI		0.14
WHO Group		0.20
WHO FC (1 or 2 vs 3 or 4)	0.21 (0.09, 0.52)	**0.003**
6MWT (per 1 m increase)	0.99 (0.985, 0.996)	** < 0.001**
NT-proBNP (per 1 pg/ml increase)	1.0006 (1.0003, 1.001)	**0.0013**
PH medication		
CCB		0.30
PDE5		0.08
ERA (ERA vs no ERA)	7.1 (1.7, 30.6)	** < 0.001**
PO PCA		0.94
SQ/IV PCA (risk SQ/IV PCA vs none)	5.6 (2.3, 13.5)	** < 0.001**
CMR		
*RV*		
RV EDVi (per 1 ml/m^2^ increase)	1.011 (1.007, 1.015)	** < 0.001**
RV ESVi (per 1 ml/m^2^ increase)	1.012 (1.008, 1.017)	** < 0.001**
RV SVi		0.18
RV Mass index (per 1 g/m^2^ increase)	1.04 (1.03, 1.06)	** < 0.001**
RV Mass: RVEDV		0.89
RV EF (per 1% increase)	0.92 (0.89, 0.95)	** < 0.001**
RV CI (per 1 L/min/m^2^ increase)	1.4 (1.0, 1.9)	**0.029**
RV GFI (per 1% increase)	0.92 (0.90, 0.95)	** < 0.001**
*LV*		
LV EDVi		0.051
LV ESVi (per 1 ml/m^2^ increase)	1.03 (1.01, 1.05)	**0.003**
LV SVi		0.56
LV Mass index (per 1 g/m^2^ increase)	1.03 (1.01, 1.05)	**0.015**
LV EF (per 1% increase)	0.94 (0.90, 0.99)	**0.030**
LV CI		0.27
LV GFI (per 1% increase)	0.93 (0.88, 0.98)	**0.005**

Bold indicates p < 0.05

Hazard ratios only reported for comparisons with p values < 0.05. *CAO* composite adverse outcome, *CCB* calcium channel blocker, *CI*  cardiac index, *CMR* cardiac magnetic resonance imaging, *EDV* end diastolic volume, *EDVi* indexed end diastolic volume, *EF* ejection fraction, *ESVi* indexed end systolic volume, *ERA* endothelin receptor antagonist, *FC* functional class, *GFI* global function index, *LV* left ventricle, *PCA* prostacyclin, *PDE5* phosphodiesterase-5 inhibitor, *PH* pulmonary hypertension, *PO* oral, *SQ/IV* parenteral, *RV* right ventricle, *SVi*  indexed stroke volume

**Fig. 2:**
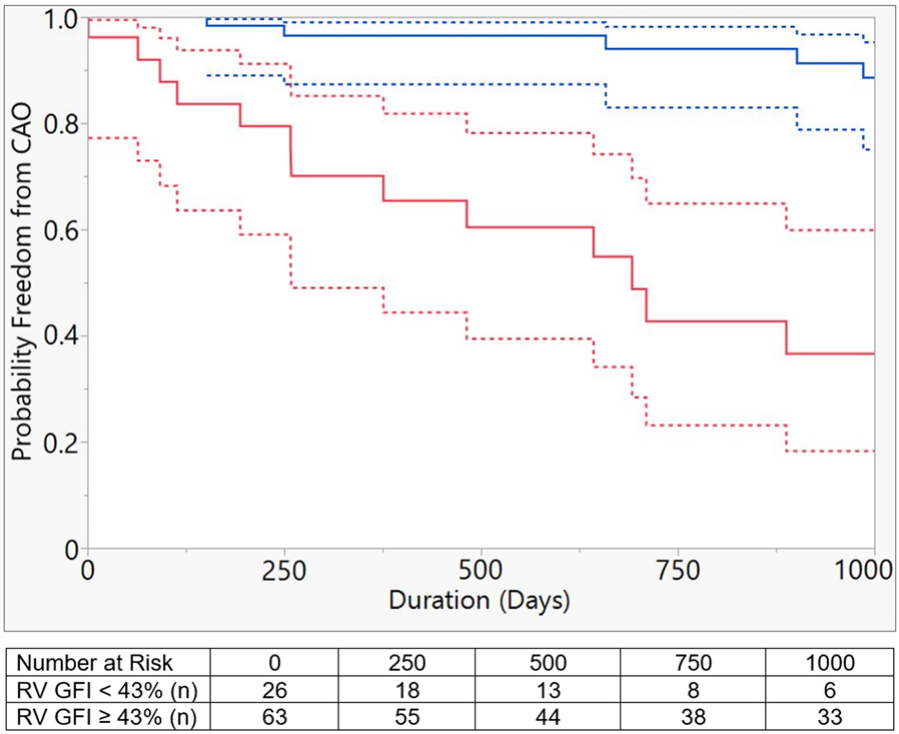
Patients were dichotomized using logistic regression threshold RV GFI of 43% (AUC 83%, 76% Sensitivity, 83% Specificity). Kaplan–Meier curve demonstrating difference in probability of freedom from composite adverse outcome (CAO: death, lung transplant, Potts shunt, or SQ/IV prostacyclin initiation) based on RV GFI ≥ 43% (blue line) and < 43% (red line) over 1000 days (median follow-up time 710 days). Dashed lines represent 95% confidence intervals (p < 0.0001)

By logistic regression, an RV GFI cut off value of 43% predicted incidence of CAO with a sensitivity of 76% and specificity of 83% (AUC 0.83). Based on this dichotomy, patients with RV GFI ≤ 43% demonstrated decreased freedom from CAO during median follow up of 2.7 years (p < 0.001) (Fig. 2).

#### Sub-analysis: mortality vs. no mortality

Of the four patients that underwent lung transplantation, two patients experienced mortality. The mortality sub-cohort comprised a total of 8 patients, which consisted of the six original patients plus the two additional patients who experienced mortality post lung transplantation. The median age at mortality was 18 years (IQR 15.2–21.3 years), and the median time to mortality was 0.78 years (IQR 0.29–3.1 years). The mortality cohort had a higher percentage of patients WHO-FC ≥ 3 (88% vs 23%, p < 0.001), decreased median 6MWT distance (346 vs. 513 m, p = 0.007), and higher median NT-proBNP levels (2460 vs. 183 pg/mL, p = 0.02) (Additional file 1: Table S2). The mortality cohort had a larger percentage of patients on SQ/IV PCA (100 vs 20%, p < 0.001) (Additional file 1: Table S2). From a CMR perspective, the mortality cohort had higher RVEDVi (p = 0.002), RV ESVi (p = 0.003), RV indexed mass (p < 0.001), and LV indexed mass (p = 0.02) (Additional file 1: Table S2). The mortality cohort had lower RV EF (p < 0.001), RV GFI (p < 0.001), and LV GFI (p = 0.028) (Additional file 1: Table S2). There were no other statistically significant differences at this sample size between the two cohorts for other CMR volumetric and functional analyses.

RV EDVi (HR = 1.01, 95% CI [1.01, 1.02]); ESVi (HR = 1.01, 95% CI [1.01, 1.02]), indexed mass (HR = 317, 95% CI [25, 5585]), LV EDVi (HR = 1.04, 95% CI [1.02, 1.08]); ESVi (HR = 1.06, 95% CI [1.03, 1.09]), and LV indexed mass (HR = 1.06, 1.03, 1.09]) were positively associated with the time to mortality (Additional file 1: Table S3). Conversely, RV EF (HR = 0.87, 95% CI [0.80, 0.92]), RV GFI (HR = 0.88, 95% CI [0.81, 0.93]), LV EF (HR = 0.04, 95% CI [0.85, 0.96]), and LV GFI (HR = 0.84, 95% CI [0.77, 0.91]) were inversely associated with the time to mortality.

#### Sub-analysis: WHO Group I sub-cohort

The WHO Group 1 sub-cohort consisted of 75 patients (Additional file 1: Table S4). Fifty-three percent of the sub-cohort was female. Twenty (27%) patients experienced a CAO during a median follow up time of 1.2 years. The median age at CAO was 18 years (IQR 10.3–18.8 years), and the median time to CAO was 1.2 years (IQR 0.44–2.3 years). When comparing those experienced CAO versus those who did not, the CAO sub-cohort had a higher percentage of patients WHO-FC ≥ 3 (60% vs 20%, p < 0.001) and decreased median 6MWT distance (412 vs. 535 m, p = 0.02) (Additional file 1: Table S4). The CAO sub-cohort had a larger percentage of patients on ERA and SQ/IV PCA (90 vs 65%, p = 0.04 and 100 vs 20%, p < 0.001, respectively) (Additional file 1: Table S4). From a CMR perspective, the CAO sub-cohort had higher RVEDVi (p < 0.001), RV ESVi (p = 0.006, and RV indexed mass (p = 0.005) (Additional file 1: Table S4). The CAO sub-cohort had lower RV EF (p < 0.001) and RV GFI (p < 0.001) (Additional file 1: Table S4). There were no other statistically significant differences at this sample size between the two cohorts for other CMR volumetric and functional analyses.

RV EDVi (HR = 1.01, 95% CI [1.01, 1.02]); ESVi (HR = 1.01, 95% CI [1.01, 1.02]), indexed mass (HR = 1.04, 95% CI [1.03, 1.06]), LV ESVi (HR = 1.03, 95% CI [1.01, 1.05]), and LV indexed mass (HR = 1.03, 1.001, 1.05]) were positively associated with the time to CAO (Additional file 1: Table S5). Conversely, RV EF (HR = 0.92, 95% CI [0.90, 0.95]), RV GFI (HR = 0.93, 95% CI [0.91, 0.96]) were inversely associated with the time to CAO.

#### Multivariable modeling

For multivariable modeling, the baseline “CMR” and “clinical” models yielded optimism corrected C-statistics of 0.83 and 0.75, respectively (Table 5). Addition of RV GFI to either baseline model yielded modest improvements in rank order discrimination as measured by the C-statistic and improvements in model fit as measured by the LRT.

**Table 5:** Multivariate models predicting CAO

Model	C-Statistic	LRT p-value
*RV EDVi, ESVi, Mass index*	0.83	
+ RV CI	0.80	0.64
+ RV EF	0.83	**0.019**
+ RV GFI	0.84	**0.032**
*WHO-FC, ERA, SQ/IV PCA*	0.75	
+ RV EDVi, ESVi, Mass index	0.76	**0.022**
+ RV EF	0.79	**0.004**
+ RV GFI	0.82	**0.002**

Bold indicates p < 0.05

*CAO* composite adverse outcome, *CI* cardiac index, *EDVi* indexed end diastolic volume, *ERA* endothelin receptor antagonist, *EF* ejection fraction, *ESVi* indexed end systolic volume, *FC* functional class, *GFI* global function index, *PCA* prostacyclin, *PO* oral, *RV* right ventricle, *SQ/IV* parenteral

#### CMR associations with secondary outcomes

RV EDVi, ESVi, and indexed mass had modest negative correlations, while RV EF and GFI had modest positive correlations with 6MWT distance (Additional file 1: Table S1). There were no correlations between LV CMR variables and 6MWT distance. There were no associations between CMR variables and a 6MWT distance less than 352 m. For NT-proBNP levels, RV EDVi, ESVi, and indexed mass had strong, positive correlations, while RV EF, and GFI had modest, negative correlations with NT-proBNP (Additional file 1: Table S1). LV ESVi had weak positive correlation; mass indexed had a modest, positive correlation; and LV GFI had modest, negative correlation with NT-proBNP levels (Additional file 1: Table S1).

## Discussion

This study is the first to evaluate ventricular GFI in pediatric PH patients. Lower CMR derived RV GFI was able to accurately identify subjects who would ultimately have CAO during the follow up period (sensitivity 76%, specificity 83%, AUC 0.83 using RV GFI cutoff of 43%). In survival analysis, subjects with RV GFI less than 43% demonstrated impaired event-free survival and increased hazard of CAO compared to those with RV GFI greater than 43%. In multivariable testing, RV GFI demonstrated improved prognostic value beyond known clinical risk factors or classically used MRI-derived markers of ventricular mass and volume. Further, determination of CMR derived ventricular GFI was feasible and easily performed in this pediatric cohort, using readily available CMR data without requiring additional post-processing.

Prior reports have demonstrated the prognostic value of CMR derived RV size and function in pediatric and adult PH patients, with RV EF demonstrating the strongest association with clinical outcomes [4, 5]. Similarly, increased RV volumes, increased mass, and decreased EF and ventricular GFI were associated with increased risk of CAO in the current study. In the multivariable models, addition of RV EF and RV GFI provided additional prognostic information compared to models that only considered RV volume and mass, consistent with previous data supporting the importance of RV function in predicting clinical outcomes in PH.

The addition of RV GFI to the baseline “CMR” multivariable model that incorporated only traditional CMR volumetric markers demonstrated improved model fit, as evidenced by the likelihood ratio test results. We elected not to include both EF and GFI in the same model as a direct comparison due to a high degree of collinearity between the variables. However, the higher C-statistic seen with addition of GFI to the baseline model compared to addition of EF or CI suggests that RV GFI may have additive value beyond the other metrics evaluated. Further studies will be needed to explore whether a specific GFI value versus change in GFI over time is more associated with CAO.

Preserved RV function in the setting of increased RV afterload requires adaptive remodeling to maintain ventricular vascular coupling. RV GFI is a marker that incorporates both ventricular function and remodeling, which may be more reflective of overall myocardial health than metrics that consider only one or the other. Badagliaca et al. [7] hypothesized that concentric hypertrophy, determined by RV mass to end diastolic volume ratio, was indicative of an adaptive RV remodeling response to increased afterload as opposed to maladaptive eccentric hypertrophy, which would be associated with high end diastolic volume in addition to high mass. As a metric that includes measurements of ejection, dilation, and hypertrophy, GFI may be sensitive to identify these variable RV responses to increased afterload, which could explain its significant prognostic capacity. For example, a patient with high RV mass but normal RV volume would have a higher GFI than one with high mass and high volume. While we did not appreciate an association between a decreased RV mass to end diastolic volume ratio and incidence of CAO in the current study, this could be due to a younger cohort and need for accrued time dealing with a significant pressure load to exhibit difference is remodeling. Nevertheless, the integration of mass into the GFI calculation may allow for longitudinal evaluation for adverse RV remodeling.

Moledina and colleagues have previously reported an association of lower LVSVi with risk of CAO in a pediatric PH cohort [4]. In contrast, neither LVSVi nor LVCI were associated with CAO in our cohort. This difference may be due to a combination of differences in baseline therapy and CAO definition, as our definition included the initiation of PCA. Sixty-four percent of the Moledina cohort was on combination therapy. While Moledina did not specifically describe prostacyclin use, 16% of patients were on no PH therapy, 34% were on monotherapy, and 48% on combination therapy at time of CMR. In contrast, in the current study 8% were on no PH therapy, 16% were on monotherapy, 76% on combination therapy, and 45% were on prostacyclin therapy at time of CMR. These differences in cohort characteristics could possibly explain the differences in association.

Higher RVCI was associated with increased risk of CAO in the current study. We hypothesize this relationship may be confounded by prostacyclin therapy in the current cohort, as prostacyclin medications are known to cause increased cardiac output and are indicated for patients with the most severe PH [18, 19]. As stated previously, while the Moledina cohort had 48% on combination therapy at time of CMR, in the current study 76% were on combination therapy, and 45% were on prostacyclin therapy at time of CMR. Therefore, the increased degree of illness and consequent increased medication requirement in our cohort may explain the discrepant findings related to RV CI as a risk factor for CAO.

### Limitations

There are several limitations with our study including those inherent to the retrospective nature of our study. First, there are no published normative values for RV GFI either in a healthy population or in a PH pediatric or adult population. Additional studies will be needed to define normative values in these populations, confirm these associations in larger cohorts, and determine cut off values that could guide clinical decision making. Second, entry point into the cohort was based on timing of clinical CMR rather than at diagnosis so some subjects had been previously treated with targeted therapy longer than others. Additionally, in our secondary outcomes analysis, recording 6MWT and NT-proBNP within 6 months of CMR may have a missed an interval change in clinical status, given our median time to CAO of 18 months. Furthermore, the lack of association between CMR parameters and a 6MWT distance < 352 m may be secondary to the clinical characteristics of our cohort with median 6MWT distances of 498 and 395 m for our total cohort and CAO sub-cohort, respectively. Given patient age, other clinical indications, or bundling of procedures, some patients may have received anesthesia during CMR, which may have affected some CMR assessments of function. Finally, our cohort also includes a moderate amount of heterogeneity in disease severity, underlying diagnosis, and treatment, which could affect results.

## Conclusion

We report that RV GFI, a novel metric that combines assessments of systolic function, ventricular dilation, and ventricular hypertrophy, was readily measurable without specialized post-processing in 100% of children with PH undergoing CMR in our multicenter cohort. On univariate testing, this metric is associated with increased hazard of CAO. With multivariable modeling, we report that RV GFI is additive to conventional CMR metrics of ventricular performance. Therefore, we propose that RV GFI may be clinically useful as a prognostic marker in pediatric PH patients beyond traditional CMR metrics.

## Supplementary Information

**Additional file 1: Table S1.** Univariate associations of CMR Variables to 6MWT and NT-proBNP. **Table S2.** Demographic and CMR data for study cohort and death sub-cohort. **Table S3.** Univariate Cox analysis for death. **Table S4.** Demographic and CMR data for WHO group 1 sub-cohort. **Table S5.** Univariate Cox Analysis with CAO, WHO Group I.

## Data Availability

The dataset used and analyzed during the study are available from the corresponding author upon reasonable request.

## Abbreviations

6MWT: Six-minute walk test
CAO: Composite adverse outcome
CI: Cardiac index
CMR: Cardiac magnetic resonance imaging
EF: Ejection fraction
ERA: Endothelin receptor antagonists
GFI: Global function index
LV: Left ventricle/left ventricular
LVEDVi: Left ventricular end diastolic volume indexed to body surface area
LVESVi: Left ventricular end systolic volume indexed to body surface area
LVSVi: Left ventricular stroke volume indexed to body surface area
NT-proBNP: Natriuretic peptide levels
PCA: Parenteral prostacyclin
PH: Pulmonary hypertension
RV: Right ventricle/right ventricular
RVEDVi: Right ventricular end diastolic volume indexed to body surface area
RVESVi: Right ventricular end systolic volume indexed to body surface area
WHO-FC: World Health Organization functional class
