# Supplementary material for: Ventricular global function index is associated with clinical outcomes in pediatric pulmonary hypertension
Source: J Cardiovasc Magn Reson. 2023 Jul 3;25:39. doi: 10.1186/s12968-023-00947-8 (PMC10316558; doi:10.1186/s12968-023-00947-8)
Supplement: Supplementary file 1 — Additional file 1: Table S1. Univariate associations of CMR Variables to 6MWT and NT-proBNP. Table S2. Demographic and CMR data for study cohort and death sub-cohort. Table S3. Univariate Cox analysis for death. Table S4. Demographic and CMR data for WHO group 1 sub-cohort. Table S5. Univariate Cox Analysis with CAO, WHO Group I. [file 12968_2023_947_MOESM1_ESM.docx]

Table S1: Univariate associations of CMR Variables to 6MWT and NT-proBNP.

|  | R or ρ | p value |
| --- | --- | --- |
| 6MWT | | |
| *RV* |  |  |
| RV EDVi | -0.35 | **0.008** |
| RV ESVi | -0.35 | **0.007** |
| RVSVi | 0.03 | 0.8 |
| RV Mass I | -0.36 | **0.006** |
| RV EF | 0.43 | **<0.001** |
| RV CI | -0.10 | 0.4 |
| RV GFI | 0.39 | **0.002** |
| *LV* |  |  |
| LV EDVi | 0.14 | 0.3 |
| LV ESVi | 0.0 | 0.98 |
| LV Svi | 0.30 | 0.02 |
| LV Mass index | -0.08 | 0.6 |
| LV EF | 0.05 | 0.7 |
| LV CI | -0.03 | 0.8 |
| LV GFI | 0.18 | 0.2 |
|  |  |  |
| NT-proBNP | | |
| *RV* |  |  |
| RV EDVi | 0.72 | **<0.001** |
| RV ESVi | 0.73 | **<0.001** |
| RV SVi | 0.21 | 0.11 |
| RV Mass I | 0.72 | **<0.001** |
| RV EF | -0.59 | **<0.001** |
| RV CI | 0.19 | 0.15 |
| RV GFI | -0.59 | **<0.001** |
| *LV* |  |  |
| LV EDVi | 0.17 | 0.2 |
| LV ESVi | 0.26 | **0.045** |
| LV SVi | -0.07 | 0.6 |
| LV Mass index | 0.42 | **<0.001** |
| LV EF | -0.23 | 0.08 |
| LV CI | 0.16 | 0.2 |
| LV GFI | -0.35 | **0.007** |

Abbreviations: 6MWT: six-minute walk test, CI = cardiac index, CMR: cardiac magnetic resonance imaging, EDV: end diastolic volume, EDVi: indexed end diastolic volume; EF: ejection fraction, ESVi: indexed end systolic volume, GFI: global function index, LV: left ventricle, NT-proBNP: N-terminal pro-hormone brain natriuretic peptide, RV: right ventricle, SVi = indexed stroke volume.

Table S2: Demographic and CMR data for study cohort and death sub-cohort

|  | **Cohort**  **(N = 89)** | **Death**  **(N = 8)** | **No Death**  **(N = 81)** | **p-value** |
| --- | --- | --- | --- | --- |
| Female n (%) | 48 (54) | 2 (25) | 46 (57) | 0.14 |
| WHO Group |  |  |  | 0.24 |
| 1 n (%) | 75 (84) | 8 (100) | 67 (83) |  |
| 3 n (%) | 13 (15) | 0 (0) | 13 (16) |  |
| 5 n (%) | 1 (1) | 0 (0) | 1 (1) |  |
| WHO FC |  |  |  | **<0.001** |
| N (%) | 86 (97) |  |  |  |
| 1 or 2 | 60 (70) | 1 (13) | 59 (73) |  |
| 3 or 4 | 26 (30) | 7 (88) | 19 (23) |  |
| 6MWT |  |  |  |  |
| n (%) | 58 (65) | 8 (100) | 50 (62) |  |
| Meters (IQR) | 498 (394 – 569) | 346 (301 - 428) | 513 (425 - 573) | **0.007** |
| NT-proBNP |  |  |  |  |
| n (%) | 58 (65) | 4 (50) | 54 (67) |  |
| pg/mL (IQR) | 191 (63 – 699) | 2460 (362 – 5080) | 183 (62 – 526) | **0.02** |
|  |  |  |  |  |
| PH medication (n, %) |  |  |  |  |
| CCB | 9 (10) | 0 (0) | 9 (11) | 1 |
| PDE5 | 73 (82) | 6 (75) | 67 (83) | 0.63 |
| ERA | 57 (64) | 7 (88) | 50 (62) | 0.25 |
| PO PCA | 16 (18) | 0 (0) | 16 (20) | 0.34 |
| SQ/IV PCA | 24 (27) | 8 (100) | 16 (20) | **<0.001** |
| Dual Therapy | 31 (35) | 3 (38) | 28 (35) |  |
| Triple Therapy | 34 (38) | 5 (63) | 29 (36) | 0.25 |
|  |  |  |  |  |
| Age at CMR (years, IQR) | 12.0 (8.0 – 17.0) | 16.5 (12.9 – 18.0) | 11.3 (8.0 – 16.0) | 0.1 |
|  |  |  |  |  |
| RV |  |  |  |  |
| RV EDVi (mL/m^2^) | 109 (89 – 135) | 200 (134 – 409) | 104 (84 – 127) | **0.002** |
| RV ESVi (mL/m^2^) | 55 (40 – 77) | 142 (89 – 360) | 52 (38 - 68) | **0.002** |
| RV SVI (mL/m^2^) | 51 (42 – 61) | 46 (42 – 72) | 51 (43 – 61) | 0.86 |
| RV Mass index (gm/m^2^) | 28 (19 – 37) | 50 (33 – 106) | 26 (19 – 34) | **<0.001** |
| RV EF (%) | 49 (43 – 56) | 26 (13 – 43) | 50 (44 – 56) | **<0.001** |
| RV CI (L/min/m^2^) | 4.2 (3.4 – 4.9) | 3.8 (2.9 – 5.8) | 4.2 (3.4 – 4.9) | 0.53 |
| RV GFI (%) | 50 (42 – 57) | 21 (10 – 38) | 51 (43 – 58) | **<0.001** |
| LV |  |  |  |  |
| LV EDVi (mL/m^2^) | 83 (70 – 96) | 108 (64 – 153) | 81 (70 – 92) | 0.12 |
| LV ESVi (mL/m^2^) | 35 (27 – 44) | 47 (26 – 113) | 35 (27 – 43) | 0.15 |
| LV SVI (mL/m^2^) | 47 (40 – 54) | 41 (29 – 58) | 47 (40 – 53) | 0.60 |
| LV Mass index (gm/m^2^) | 42 36 – 54) | 65 (43 - 100) | 42 (35 – 51) | **0.02** |
| LV EF (%) | 56 54 – 61) | 56 (30 – 63) | 56 (54 – 61) | 0.49 |
| LV CI (L/min/m^2^) | 3.7 (3.0 – 4.5) | 3.6 (2.1 – 5.8) | 3.7 (3.0 – 4.4) | 0.82 |
| LV GFI (%) | 46 (41 – 51) | 36 (23 – 43) | 47 (41 – 52) | **0.004** |

Data are median (Interquartile range). P-values obtained from independent samples t, Fisher’s exact, or Wilcoxon rank-sum test between death and no death groups. Abbreviations: 6MWT: six-minute walking test, CCB: calcium channel blocker, CI = cardiac index, CMR: cardiac magnetic resonance imaging, EDV: end diastolic volume, EDVi: indexed end diastolic volume; EF: ejection fraction, ESVi: indexed end systolic volume, ERA: endothelin receptor antagonist, FC: functional class, GFI: global function index, LV: left ventricle, NT-proBNP: N-terminal pro-hormone brain natriuretic peptide, PCA: prostacyclin, PDE5: phosphodiesterase-5 inhibitor, PH: pulmonary hypertension, PO: oral, SQ/IV: parenteral, RV: right ventricle, SVi = indexed stroke volume, WHO: World Health Organization.

Table S3: Univariate Cox analysis for death

| **Univariate Cox analysis** | **Hazard ratio (95% CI)** | **p value** |
| --- | --- | --- |
| Gender |  | 0.43 |
| Age at MRI (per 1 year increase) | 1.2 (1.05, 1.56) | **0.01** |
| WHO Group |  | 0.24 |
| WHO FC (1 or 2 vs 3 or 4) | 0.04 (0.004, 0.31) | **0.002** |
| 6MWT (per 1 meter increase) | 0.98 (0.97, 0.99) | **<0.001** |
| NT-proBNP (per 1 pg/ml increase) | 147 (6, 10950) | **0.003** |
|  |  |  |
| PH medication |  |  |
| CCB |  | 0.12 |
| PDE5 |  | 0.98 |
| ERA (ERA vs no ERA) |  | 0.07 |
| PO PCA |  | 0.12 |
| SQ/IV PCA (risk SQ/IV PCA vs none) |  | 0.9 |
|  |  |  |
| CMR |  |  |
| *RV* |  |  |
| RV EDVi (per 1 ml/m^2^ increase) | 1.01 (1.007, 1.019) | **<0.001** |
| RV ESVi (per 1 ml/m^2^ increase) | 1.01 (1.009, 1.023) | **<0.001** |
| RV SVi |  | 0.59 |
| RV Mass index (per 1 gm/m^2^ increase) | 317 (25, 5585) | **<0.001** |
| RV Mass: RVEDV |  | 0.96 |
| RV EF (per 1% increase) | 0.87 (0.80, 0.92) | **<0.001** |
| RV CI (per 1 L/min/m^2^ increase) |  | 0.56 |
| RV GFI (per 1% increase) | 0.88 (0.81, 0.93) | **<0.001** |
|  |  |  |
| *LV* |  |  |
| LV EDVi (per 1 ml/m^2^ increase) | 1.04 (1.016, 1.075) | **0.002** |
| LV ESVi (per 1 ml/m^2^ increase) | 1.06 (1.032, 1.088) | **<0.001** |
| LV SVi |  | 0.35 |
| LV Mass index (per 1 gm/m^2^ increase) | 1.06 (1.028, 1.094) | **<0.001** |
| LV EF (per 1% increase) | 0.90 (0.85, 0.96) | **0.002** |
| LV CI |  | 0.94 |
| LV GFI (per 1% increase) | 0.84 (0.77, 0.91) | **<0.001** |

Hazard ratios only reported for comparisons with p values < 0.05. Abbreviations:

6MWT: six-minute walking test, CCB: calcium channel blocker, CI = cardiac index, CMR: cardiac magnetic resonance imaging, EDV: end diastolic volume, EDVi: indexed end diastolic volume; EF: ejection fraction, ESVi: indexed end systolic volume, ERA: endothelin receptor antagonist, FC: functional class, GFI: global function index, LV: left ventricle, NT-proBNP: N-terminal pro-hormone brain natriuretic peptide, PCA: prostacyclin, PDE5: phosphodiesterase-5 inhibitor, PH: pulmonary hypertension, PO: oral, SQ/IV: parenteral, RV: right ventricle, SVi = indexed stroke volume, WHO: World Health Organization.

Table S4: Demographic and CMR data for WHO group 1 sub-cohort

|  | **WHO I Cohort (N=75)** | **CAO**  **(N=20)** | **No CAO**  **(N=55)** | **p-value** |
| --- | --- | --- | --- | --- |
| Female n (%) | 40 (53) | 9 (45) | 31 (56) | 0.44 |
|  |  |  |  |  |
| WHO FC |  |  |  | **<0.001** |
| N (%) | 72 (96) | 19 (95) | 53 (96) |  |
| 1 or 2 | 49 (65) | 7 (35) | 42 (76) |  |
| 3 or 4 | 23 (31) | 12 (60) | 11 (20) |  |
| 6MWT |  |  |  | **0.02** |
| n (%) | 55 (73) | 18 (90) | 37 (67) |  |
| Meters (IQR) | 498 (390 – 568) | 412 (330 – 539) | 535 (442 – 575) |  |
| NT-proBNP |  |  |  | 0.058 |
| n (%) | 52 (69) | 15 (75) | 37 (67) |  |
| pg/mL (IQR) | 206 (62 – 766) | 517 (208 – 2160) | 128 (54 – 421) |  |
|  |  |  |  |  |
| PH medication (n, %) |  |  |  |  |
| CCB | 7 (9) | 1 (5) | 6 (11) | 0.7 |
| PDE5 | 65 (87) | 18 (90) | 47 (85) | 1 |
| ERA | 54 (72) | 18 (90) | 36 (65) | **0.04** |
| PO PCA | 16 (21) | 3 (15) | 13 (24) | 0.53 |
| SQ/IV PCA | 23 (31) | 13 (65) | 10 (18) | **<0.001** |
| Dual Therapy | 28 (37) | 7 (35) | 21 (38) | 1 |
| Triple Therapy | 33 (44) | 13 (65) | 20 (36) | **0.036** |
|  |  |  |  |  |
| Age at CMR, years (IQR) | 13.3 (10 – 17) | 13.0 (10.0 – 17.0) | 13.3 (10.0 – 17.0) | 0.9 |
|  |  |  |  |  |
| RV |  |  |  |  |
| RV EDVi (mL/m^2^) | 115 (93 – 145) | 146 (121 - 245 | 105 (92 – 123) | **<0.001** |
| RV ESVi (mL/m^2^) | 60 (43 – 84) | 89 (63 – 196) | 53 (42 – 69) | **0.006** |
| RV SVI (mL/m^2^) | 52 (45 – 62) | 56 (42 – 64) | 52 (45 – 61) | 0.60 |
| RV Mass index (gm/m^2^) | 28 (19 – 37) | 37 (31 – 59) | 24 (19 – 32) | **0.005** |
| RV EF (% ) | 48 (42 – 54) | 40 (20 – 46) | 49 (44 – 56) | **<0.001** |
| RV CI (L/min/m^2^) | 4.2 (3.4 – 4.9) | 4.7 (3.5 – 5.6) | 4.1 (3.3 – 4.6) | 0.20 |
| RV GFI (% ) | 49 (38 - 57) | 38 (17 – 43) | 52 (44 – 59) | **< 0.001** |
| LV |  |  |  |  |
| LV EDVi (mL/m^2^) | 85 (74 – 100) | 86 (68 – 111) | 85 (76 – 100) | 0.4 |
| LV ESVi (mL/m^2^) | 36 (31 – 45) | 35 (27 – 51) | 37 (31 – 44) | 0.18 |
| LV SVi (mL/m^2^) | 48 (42 – 56) | 43 (38 – 56) | 48 (42- 55) | 0.19 |
| LV Mass index (gm/m^2^) | 44 (37 – 55) | 44 (40 – 57) | 44 (36 – 54) | 0.17 |
| LV EF (% ) | 56 (53 – 60) | 57 (49 – 62) | 56 (54 – 60) | 0.20 |
| LV CI (L/min/m^2^) | 3.8 (3.0 – 4.5) | 4.0 (3.0 – 4.9) | 3.7 (3.0 – 4.4) | 0.40 |
| LV GFI (%) | 46 (41 – 51) | 42 (37 – 49) | 47 (42 – 51) | 0.059 |

Data are median (Interquartile range). P-values obtained from independent samples t, Fisher’s Exact, or Wilcoxon rank-sum test between death and no death groups. Abbreviations: 6MWT: six-minute walking test, CCB: calcium channel blocker, CI = cardiac index, CMR: cardiac magnetic resonance imaging, EDV: end diastolic volume, EDVi: indexed end diastolic volume; EF: ejection fraction, ESVi: indexed end systolic volume, ERA: endothelin receptor antagonist, FC: functional class, GFI: global function index, LV: left ventricle, NT-proBNP: N-terminal pro-hormone brain natriuretic peptide, PCA: prostacyclin, PDE5: phosphodiesterase-5 inhibitor, PH: pulmonary hypertension, PO: oral, SQ/IV: parenteral, RV: right ventricle, SVi = indexed stroke volume, WHO: World Health Organization.

Table S5: Univariate Cox Analysis with CAO, WHO Group I

| **Univariate Cox analysis** | **Hazard ratio (95% CI)** | **p value** |
| --- | --- | --- |
| Gender |  | 0.6 |
| Age at MRI |  | 0.7 |
| WHO FC (1 or 2 vs 3 or 4) | 0.17 (0.07, 0.44) | **<0.001** |
| 6MWT (per 1 meter increase) | 0.99 (0.986, 0.996) | **<0.001** |
| NT-proBNP (per 1 pg/ml increase) | 1.0006 (1.0002, 1.0009) | **<0.001** |
|  |  |  |
| PH medication |  |  |
| CCB |  | 0.23 |
| PDE5 |  | 0.28 |
| ERA (ERA vs no ERA) | 5.1 (1.2, 22.1) | **0.008** |
| PO PCA |  | 0.7 |
| SQ/IV PCA (risk SQ/IV PCA vs none) | 5.2 (2.1, 13.1) | **<0.001** |
|  |  |  |
| CMR |  |  |
| *RV* |  |  |
| RV EDVi (per 1 ml/m^2^ increase) | 1.01 (1.006, 1.015) | **<0.001** |
| RV ESVi (per 1 ml/m^2^ increase) | 1.01 (1.007, 1.016) | **<0.001** |
| RV SVi |  | 0.55 |
| RV Mass I (per 1 g/m^2^ increase) | 1.04 (1.025, 1.058) | **<0.001** |
| RV Mass: RVEDV | 12985 (4.6, 20915413) | **0.02** |
| RV EF (per 1% increase) | 0.92 (0.90, 0.95) | **<0.001** |
| RV CI (per 1 L/min/m^2^ increase) |  | 0.06 |
| RV GFI (per 1% increase) | 0.93 (0.91, 0.96) | **<0.001** |
|  |  |  |
| *LV* |  |  |
| LV EDVi |  | 0.25 |
| LV ESVi (per 1 ml/m^2^ increase) | 1.03 (1.007, 1.049) | **0.012** |
| LV SVi |  | 0.10 |
| LV Mass I (per 1 g/m^2^ increase) | 1.027 (1.001, 1.051) | **0.015** |
| LV EF (per 1% increase) |  | 0.053 |
| LV CI |  | 0.36 |
| LV GFI (per 1% increase) | 0.93 (0.89, 0.98) | **0.006** |

Hazard ratios only reported for comparisons with p values < 0.05. Abbreviations: 6MWT: six-minute walking test, CCB: calcium channel blocker, CI = cardiac index, CMR: cardiac magnetic resonance imaging, EDV: end diastolic volume, EDVi: indexed end diastolic volume; EF: ejection fraction, ESVi: indexed end systolic volume, ERA: endothelin receptor antagonist, FC: functional class, GFI: global function index, LV: left ventricle, NT-proBNP: N-terminal pro-hormone brain natriuretic peptide, PCA: prostacyclin, PDE5: phosphodiesterase-5 inhibitor, PH: pulmonary hypertension, PO: oral, SQ/IV: parenteral, RV: right ventricle, SVi = indexed stroke volume, WHO: World Health Organization.
